# Reduced-intensity, risk factor–stratified immunosuppression for acquired hemophilia A: single-center observational study

**DOI:** 10.1007/s00277-020-04150-y

**Published:** 2020-07-03

**Authors:** Christiane Dobbelstein, Georgios Leandros Moschovakis, Andreas Tiede

**Affiliations:** Department of Hematology, Hemostasis, Oncology, and Stem Cell Transplantation, Hannover Medical School, Carl Neuberg Str. 1, 30625 Hannover, Germany

**Keywords:** Factor VIII, Inhibitor, Steroids, Dexamethasone, Rituximab, Cyclophosphamide

## Abstract

Immunosuppressive therapy (IST) is administered to patients with acquired hemophilia A (AHA) to eradicate autoantibodies against coagulation factor VIII (FVIII). Data from registries previously demonstrated that IST is often complicated by adverse events, in particular infections. This pilot study was set out to assess the feasibility of reduced-intensity, risk factor–stratified IST. We followed a single-center consecutive cohort of twenty-five patients with AHA receiving IST according to a new institutional treatment standard. Based on results from a previous study, GTH-AH 01/2020, patients were stratified into “poor risk” (FVIII < 1 IU/dl or inhibitor ≥ 20 Bethesda units (BU)/ml) or “good risk” (FVIII ≥ 1 IU/dl and inhibitor < 20 BU/ml). Outcomes were compared between the current cohort and the GTH registry as a historic control (*n* = 102). Baseline characteristics of the cohort were not different from the historic control. Partial remission, defined as FVIII recovered to > 50 IU/dl, was achieved by 68% of patients after a median time of 112 days, which was lower and significantly later than in the historic control (hazard ratio: 1.8, 95% confidence interval 1.2–2.8). Complete remission, overall survival, and frequency of fatal infections were not different. Grade 3 and 4 infections were more frequent. The impact of risk factors that was observed in the historic cohort was no longer apparent, as partial and complete remission and overall survival were similar in “good risk” and “poor risk” patients. In conclusion, reduced-intensity, risk factor–stratified IST is feasible in AHA but did not decrease the risk of infections and mortality in this cohort.

## Introduction

Acquired hemophilia A (AHA) is an autoimmune disorder. Autoantibodies formed against coagulation factor VIII (FVIII) cause a severe impairment of hemostasis [1–3]. It is usually recommended to administer immunosuppressive therapy in patients with AHA to induce remission of the disease. Glucocorticoids, cyclophosphamide, and rituximab, or combinations thereof, are frequently used for immunosuppression in AHA [4].

Patients not receiving immunosuppression can also achieve remission, but this outcome is not predictable. In a single-center cohort reported in 1987, Lottenberg et al. observed spontaneous remission in 5 out of 16 patients after up to 2 years [5]. However, two patients died from hemorrhage during this time. Cases of late fatal bleeding (3 patients > 100 days after diagnosis) were also observed in the 2007 UK surveillance study [6]. These observations resulted in international recommendations to treat all patients with AHA with immunosuppressive therapy, regardless of their initial bleeding tendency, FVIII activity, or inhibitor titer [1, 7].

From 2010 to 2013, the German, Austrian and Swiss Thrombosis and Hemostasis Society (GTH) study group used a standardized regimen, that was designed based on the 2009 International Recommendation by Huth-Kühne et al. [7], and collected outcomes in a multicenter observational study [8–10]. The GTH-AH 01/2010 cohort was unique because of its prospective observational design, enrolling patients within 7 days after treatment start and securing that information about survival, remission, and adverse events were collected in all patients. Possibly as a result of its design, the mortality rate of 34% observed in this study was one of the highest ever reported in AHA. It was noted that mortality due to IST-related infection was the most frequent cause of death (16%) and exceeded the mortality from bleeding (3%) [9]. A similar observation was made in the French SASHA registry published in 2013 [11]. Based on these results, immunosuppressive regimens were identified as an important field of future research. More recent expert recommendations put more emphasis on preventing side effects by suggesting caution in frail patients [2].

The GTH study group had observed FVIII activity and inhibitor titer at the time of diagnosis as prognostic factors for remission and survival [9]. While an impact of these factors on remission is straightforward, their impact on survival was remarkable. The effect did not appear to result from increased bleeding in patients with low FVIII or high inhibitor titers, but rather from longer time of immunosuppressive drug exposure in such patients.

It was considered particularly important that patients with baseline FVIII ≥ 1 IU/dl *and* inhibitor titer < 20 BU/ml (“good risk”), comprising about one-third of the GTH cohort, had a > 50% chance of achieving PR with glucocorticoids alone within the first 3 weeks. Patients not belonging to this group (FVIII < 1 IU/dl *or* inhibitor titer ≥ 20 BU/ml, “poor risk”) had a < 10% chance of achieving PR after 3 weeks of steroids. It was therefore considered by the group to stratify patients according to prognostic factors and to administer more intense treatment in “poor risk” patients up front.

In addition, it was believed that the prolonged exposure to glucocorticoids, as implied by the original GTH protocol, could be a major cause of infection and other morbidity. Steroid pulse therapy as compared with continuous exposure could possibly result in fewer side effects while preserving efficacy. This idea was supported by data from other autoimmune disorders such as immune thrombocytopenia. A Chinese randomized controlled trial compared dexamethasone 40 mg/day for 4 days (repeated after 2 weeks in non-responders) with prednisone 1 mg/kg daily (4 weeks) [12]. Dexamethasone resulted in a significantly higher rate of initial response (82% vs. 67%) and complete response (50% vs. 27%). Dexamethasone pulse therapy was also associated with less frequent infection (0% vs. 3%), body weight gain (0% vs. 10%), edema (0% vs. 4%), and Cushingoid appearance (0% vs. 13%).

**Fig. 1:**
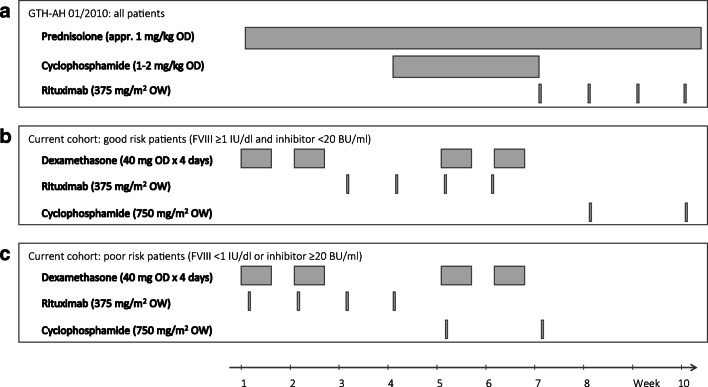
Schematic representation of immunosuppressive treatment protocols. (**a**) GTH-AH 01/2010 protocol. (**b**) Current cohort good-risk patients. (**c**) Current cohort poor-risk patients. Abbreviations: OD, once daily; OW, once weekly

Based on GTH study results and this additional information, that both became available by 2015, we designed a modified immunosuppressive regimen for AHA comprising dexamethasone pulse therapy and risk-stratified, earlier administration of rituximab (Fig. 1). The regimen was used since 2015 as an institutional treatment standard. Here we report our observations in 25 consecutive patients and compare outcomes to data from the GTH-AH 01/2010 study.

## Methods

### Study populations

#### Current cohort

The current study was a single-center, prospective, observational study of consecutive patients with AHA, defined by the presence of a neutralizing factor VIII inhibitor ≥ 0.6 Bethesda units (BU)/ml and a factor VIII activity < 50 IU/dl. All patients treated between January 2015 and July 2019 were enrolled constituting the *intention-to-treat (ITT) population*. This population was considered most comparable with the GTH study cohort (see below). Consecutive enrollment of the current cohort was ensured by screening the institutional laboratory database for all patients with positive Bethesda inhibitor tests during the time period. Patients with congenital hemophilia A with or without inhibitors were excluded. Otherwise, no inclusion or exclusion criteria were applied. In an additional analysis, only those patients receiving therapy fully adherent to the institutional standard were assessed as the *per protocol (PP) population*. The research protocol was approved by the institutional ethics committee.

#### GTH-AH 01/2010 cohort

The historic control population consisted of the multicenter, prospective, observational GTH-AH 01/2010 study enrolling patients with AHA between April 2010 and April 2013 [9]. Inclusion and exclusion criteria of this study were identical to the current study.

### Treatment

An overview on the immunosuppressive treatment regimens is provided in Fig. 1.

#### Current cohort

According to our institutional treatment standard, baseline laboratory data, obtained usually at the day of diagnosis or referral to our institution, were used to stratify patients into “good risk” (factor VIII activity ≥ 1 IU/dl *and* inhibitor < 20 BU/ml) or “poor risk” (factor VIII activity < 1 IU/dl *or* inhibitor ≥ 20 BU/ml). Treatment consisted of oral dexamethasone 40 mg per day on 4 consecutive days in weeks 1, 2, 5, and 6 (all patients); intravenous rituximab 375 mg/m^2^ body surface area (BSA) in weeks 1 to 4 (poor-risk patients) or 3 to 6 (good-risk patients); and intravenous cyclophosphamide 750 mg/m^2^ BSA in weeks 5 and 7 (poor risk) or 8 and 10 (good risk). Therapy was stopped as soon as patients achieved partial remission (see below). There was no tapering of the glucocorticoid.

#### GTH-AH 01/2010 cohort

Treatment in the GTH study was based on a consensus protocol developed in 2010. It consisted of daily oral prednisolone (75 mg for patients < 60 kg, 100 mg for 60–100 kg, 150 mg for > 100 kg body weight) in weeks 1 to 10; daily oral cyclophosphamide (100 mg for < 60 kg, 150 mg for 60–100 kg, 200 mg for > 100 kg) in weeks 4 to 6; and intravenous rituximab (375 mg/m^2^ BSA) in weeks 7 to 10.[9] Treatment escalation was withheld if a steady increase of factor VIII activity was observed during 7 days before starting cyclophosphamide or rituximab. If partial remission was achieved, cyclophosphamide and rituximab were stopped, if applicable, and prednisolone was tapered over 6 weeks (descending daily doses of 50, 25, 20, 15, 10, 5 mg for 1 week each).

### Endpoints

The definition of endpoints was identical to publications of the GTH-AH 01/2010 study:[9] Partial remission was defined as factor VIII activity restored to > 50 IU/dl and no active bleeding after stopping any hemostatic drug for > 24 h. Complete remission was defined as partial remission plus negative inhibitor test, prednisolone tapered to < 15 mg/day (only applicable for the GTH study), and any other immunosuppressive drug stopped. Additional endpoints were overall survival and frequency of grade 3 and 4 infections.

### Statistical analysis

The day of starting glucocorticoid therapy was defined as day 1 for consistency with the GTH study reports. Medians and ranges or interquartile ranges (IQR), or patient/event numbers and frequencies were used to describe data. Frequencies were compared using Fisher’s exact or chi square tests. Time to partial or complete remission and overall survival were assessed using Kaplan Meier analysis and Cox regression analysis. A *P* value < 0.05 was considered statistically significant for all analyses.

## Results

The current cohort consisted of twenty-five patients with AHA. Table 1 provides baseline characteristics of the cohort compared with the GTH-AH 01/2010 cohort, that was used as a historic control for our study. There were no significant differences between the two cohorts, although patients in the current cohort tended to be older (median age 79 vs. 74 years), were more often female (64 vs. 42%), and had more often a poor WHO performance status of score 4 or 5 (36 vs. 16%). The median follow-up time period was 269 days; the total follow-up period was 27 patient-years.

**Table 1:** Demographics and baseline characteristics of the current cohort (ITT population) as compared with the GTH-AH 01/2010 cohort [9]

Characteristic		Current cohort	GTH cohort	*P* value
		(*n* = 25)	(*n* = 102)	
Gender	Female, *n* (%)	16 (64)	43 (42)	0.072
	Male, *n* (%)	9 (36)	59 (58)	
Underlying disorder	None/idiopathic, *n* (%)	19 (76)	68 (67)	0.847
	Autoimmunity, *n* (%)	4 (16)	20 (20)	
	Malignancy, *n* (%)	2 (8)	13 (13)	
	Pregnancy, *n* (%)	1 (4)	5 (5)	
Concomitant disorders	Coronary artery disease, *n* (%)	6 (24)	28 (27)	0.806
	Heart failure, *n* (%)	7 (28)	30 (29)	1.000
	Renal failure, *n* (%)	14 (56)	37 (36)	0.110
	Arterial hypertension, *n* (%)	17 (68)	59 (58)	0.495
	Diabetes mellitus type 2, *n* (%)	7 (28)	28 (27)	1.000
WHO performance status	0, *n* (%)	4 (16)	15 (15)	0.087
	1, *n* (%)	2 (8)	26 (25)	
	2, *n* (%)	6 (24)	23 (23)	
	3, *n* (%)	4 (16)	22 (22)	
	4, *n* (%)	8 (32)	15 (15)	
	5, *n* (%)	1 (4)	1 (1)	
Age in years—median (range)		79 (35–94)	74 (26–97)	0.130
Body weight in kg—median (IQR)		70 (64–87)	77 (69–85)	0.234
Factor VIII activity in IU/dl—median (IQR)		2.8 (< 1–7)	1.4 (< 1–4)	0.080
Inhibitor in Bethesda units/ml—median (IQR)		14 (4–47)	19 (7–72)	0.208
Risk category^†^	Good risk, *n* (%)	12 (48)	37 (36)	0.360
	Poor risk, *n* (%)	13 (52)	65 (64)	

^†^Good risk: factor VIII activity ≥ 1 IU/dl and inhibitor < 20 BU/ml. Poor risk: factor VIII activity < 1 IU/dl or inhibitor ≥ 20 BU/ml

**Fig. 2:**
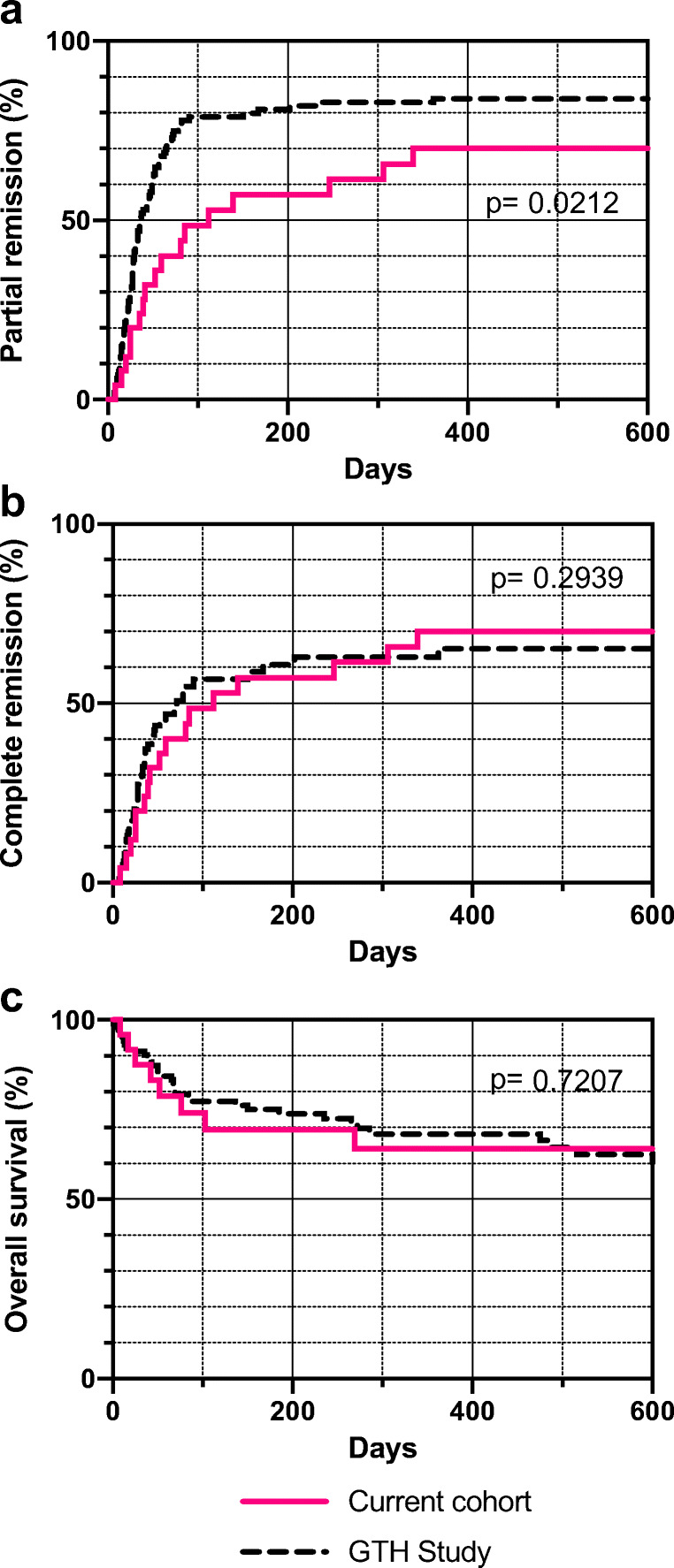
Kaplan Meier analysis of outcomes comparing the current cohort (ITT population, red solid lines) with the historic GTH-AH 01/2010 cohort (black dashed lines). (**a**) Partial remission, defined as FVIII ≥ 50%, no ongoing bleeding, and no hemostatic therapy for ≥ 24 h. (**b**) Complete remission, defined as partial remission and immunosuppressive therapy stopped (or prednisolone reduced to 15 mg/day or lower in the GTH study). (**c**) Overall survival

Treatment outcomes are summarized in Table 2 and Fig. 2. Patients of the current cohort achieved partial remission less often (68%) and later (median 112 days) as compared with patients in the historic GTH-AH 01/2010 control (83%, median 36 days). This difference was statistically significant. No difference was observed regarding complete remission or overall survival. Grade 3 or 4 infections affected more patients in the current cohort (64%) as compared with the control (36%).

**Table 2:** Main outcomes of the current cohort (ITT population) as compared with the GTH-AH 01/2010 cohort

Outcome		Current cohort	GTH cohort	*P* value
		(*n* = 25)	(*n* = 102)	
Partial remission				
	Achieved, *n* (%)	17 (68)	85 (83)	
	Median time to achievement, days (range)	112 (8–339)	36 (7–362)	
	Hazard ratio (unadjusted, 95% CI)	1.8 (1.2–2.8)		0.021
Complete remission				
	Achieved, *n* (%)	17 (68)	43 (42)	
	Median time to achievement, days (range)	112 (41–339)	71 (26–588)	
	Hazard ratio (unadjusted, 95% CI)	1.3 (0.8–2.2)		0.294
Overall survival				
	Deceased, *n* (%)	9 (36)	34 (33)	
	Hazard ratio (unadjusted, 95% CI)	0.9 (0.4–1.9)		0.721
Infections				
	Patients with grade 3 and 4 events, *n* (%)	16 (64)	37 (36)	0.014
	Patients dying from infection, *n* (%)	2 (8)	16 (16)	0.523

*P* values are derived from log-rank (Mantel-Cox) test

**Fig. 3:**
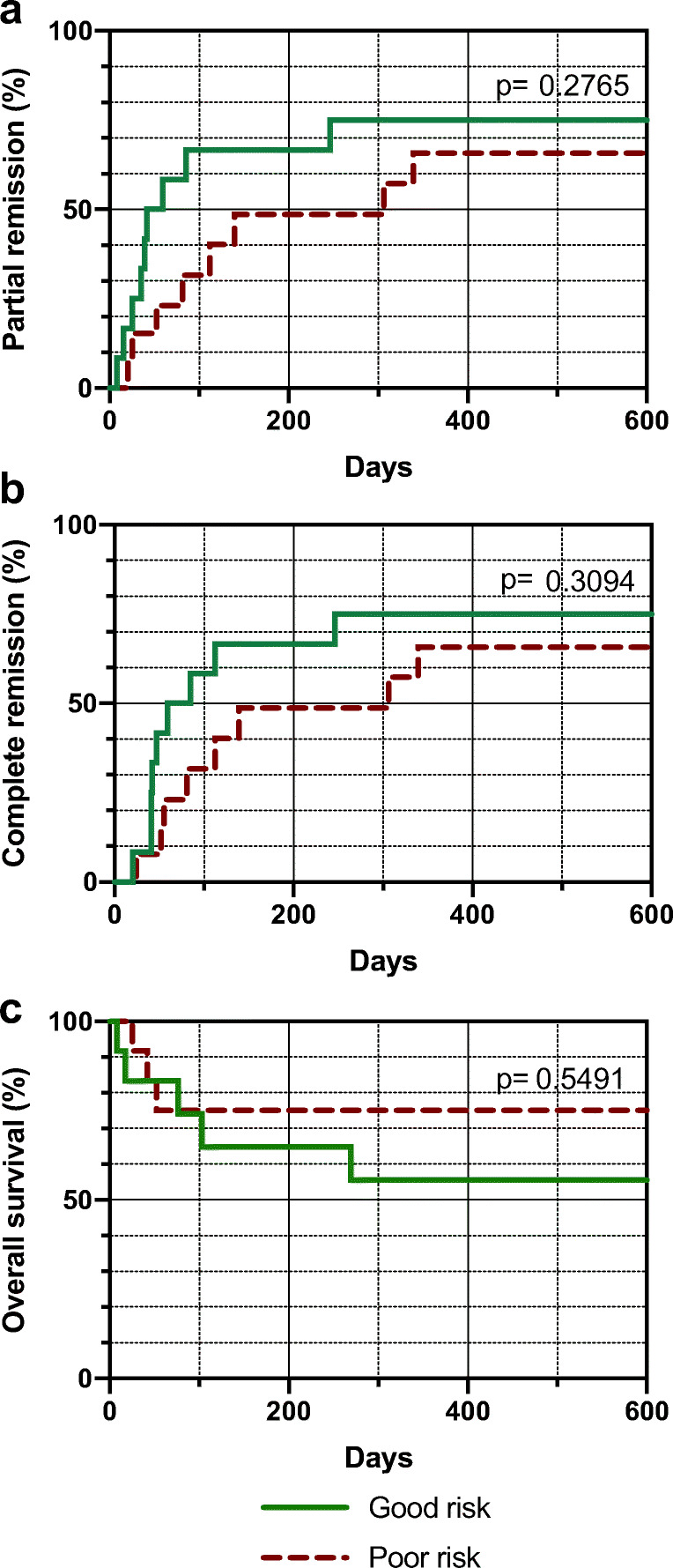
Kaplan Meier analysis comparing patients stratified to “good risk” (green solid lines) and “poor risk” (red dashed lines) of the current cohort. (**a**) Partial remission. (**b**) Complete remission. (**c**) Overall survival

Patients stratified to “poor risk” (*n* = 13, 52%) started on rituximab together with glucocorticoids, and four of them were escalated to cyclophosphamide. Patients stratified to “good risk” (*n* = 12, 48%) started on glucocorticoids alone; six of them were escalated to rituximab, and one later on to cyclophosphamide. Figure 3 shows time to partial and complete remission and overall survival according to risk category within the current cohort. The difference between “good risk” and “poor risk” patients, that had been seen in the GTH study, was not apparent in the current cohort.

**Fig. 4:**
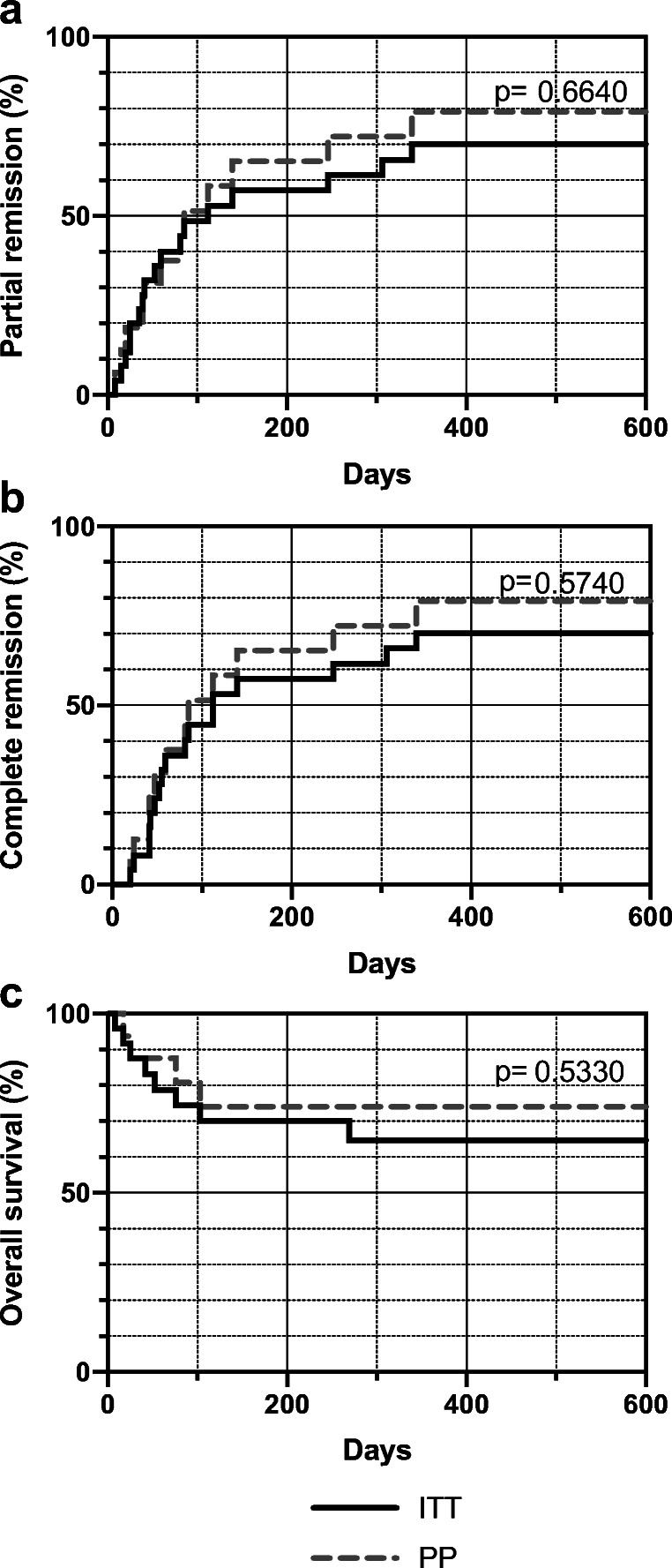
Kaplan Meier analysis comparing the intention-to-treat (ITT) and per protocol (PP) populations. (**a**) Partial remission. (**b**) Complete remission. (**c**) Overall survival

To assess the potential impact of protocol deviations, individual immunosuppressive treatments were revisited for each patient. It was found that two patients did not receive immunosuppressive therapy because they were in very poor condition. Seven patients started on prednisolone instead of dexamethasone. No deviations from the institutional standard were noted in the other patients. Figure 4 provides a comparison between the ITT and PP populations that did not show any differences in the likelihood of achieving endpoints.

## Discussion

The modified immunosuppressive regimen examined in this study differed from the GTH-AH 01/2010 protocol in several aspects:

- Dexamethasone pulse therapy instead of continuous prednisolone.
- Lower overall glucocorticoid exposure.
- Intravenous cyclophosphamide pulses instead of continuous oral cyclophosphamide.
- Rituximab before cyclophosphamide in the escalation sequence.
- Early combination therapy (steroid/rituximab) in poor-risk patients.

Stratification according to baseline prognostic factors eliminated the difference in time to remission between “good risk” and “poor risk” patients, that was previously observed in the GTH study. Although the up-front use of rituximab in “poor risk” patients can be seen as an intensification of treatment compared with the historic GTH protocol, it was probably compensated by the reduced exposure to glucocorticoids. The net effect seemed to have been a reduced intensity of immunosuppression in most patients, as supported by the longer time to achieve partial remission.

Although our study showed that reduced-intensity, risk-stratified immunosuppressive therapy is feasible, it also dampened the expectation that this approach could dramatically improve patient-relevant outcomes in AHA. We did not observe fewer infections or improved mortality. This could be explained, in part, with the overall longer exposure to immunosuppressive drugs in our patients. It should be remembered that achieving partial remission defined the end of immunosuppressive regimen, both here and in the GTH study, and that a longer time to this endpoint results in longer exposure to treatment. Furthermore, only the dosing of steroids was significantly reduced here, while the dose of rituximab was still quite high. Reduced doses of rituximab have been used without loss of efficacy in low-risk patients with autoimmune hemolytic anemia [13] and also in AHA [14].

On the other hand, several limitations of our study should be recognized. The size of our single-center cohort is limited, not surprising given the rarity of the disorder. However, we considered the present analysis as an important pilot study before implementing a modified protocol as part of future GTH multicenter study activities. Longer enrolment time was not considered feasible or useful in light of the results reported here. With the GTH-AH 01/2010 study cohort, we chose the best available control group for our analysis. However, some differences between the two cohorts were observed, including non-significant trends towards more advanced age and more frequent female gender of patients. Despite such important limitations, the rates of infection and mortality observed here are a clear indication that the current protocol cannot be expected to achieve dramatic improvements in the management of AHA. Therefore, we believe it is worthwhile to publish our data at this point in time.

The outcomes of our study should also be discussed in a broader context of AHA management. The intention of immunosuppression is generally to eliminate the risk of future bleeding by eradicating the inhibitor [15]. This is necessary because an effective hemostatic prophylaxis against bleeding has not been established. The short half-life of recombinant factor VIIa is limiting the use of this agent in prophylaxis. Activated prothrombin complex concentrate has been used for secondary prophylaxis with an apparent reduction in bleeding [16, 17]. However, this approach may require several infusions per week, probably not feasible in many AHA patients over prolonged periods of time. Recombinant porcine FVIII does not appear to be an appropriate option neither, because of de novo formation of anti-porcine inhibitors [18]. Thus, the currently used agents for treatment of acute bleeds do not seem to be very useful for long-term prophylaxis in AHA.

Non-factor replacement therapies (NFRT), recently studied in congenital hemophilia with inhibitors, could be interesting candidates for long-term prophylaxis in AHA. These include the FVIIIa-mimetic bispecific antibody emicizumab, several anti-tissue factor pathway inhibitor antibodies (concizumab, marstacimab), and the antithrombin-directed siRNA fitusiran [19]. Efficacy and safety of these treatments have been studied, or are still under investigation, in congenital hemophilia with or without inhibitors. It will not be possible to extrapolate these data to patients with AHA, who are usually much older than congenital hemophilia patients, have multiple comorbidities and poor physical performance. The use of emicizumab has occasionally been reported in AHA [20–22], and pro-hemostatic effects have been documented in ex vivo spiking studies of samples from AHA [23]. However, the slow accumulation of emicizumab with the current subcutaneous dosing regimens and its long plasma half-life create questions on the use in AHA that need to be addressed by well-designed clinical trials.

In summary, IST is still associated with significant side effects, morbidity, and mortality in patients with AHA. The reduced-intensity, stratified regimen that was examined in our single-center observational study appears feasible and resulted in similar results comparing “good risk” and “poor risk” patients. Compared with historic data from the GTH study, our regimen resulted in prolonged time to partial remission but otherwise similar outcomes. Therefore, a significant medical need remains to reduce the risk of bleeding in AHA without the burden of current immunosuppressive regimens.
